# Characterization of a conductive hydrogel@Carbon fibers electrode as a novel intraneural interface

**DOI:** 10.1186/s42234-024-00154-5

**Published:** 2024-08-27

**Authors:** Alice Giannotti, Ranieri Santanché, Ciro Zinno, Jacopo Carpaneto, Silvestro Micera, Eugenio Redolfi Riva

**Affiliations:** 1 The Biorobotic Institute, Scuola Superiore Sant’Anna, Piazza Martiri Della Libertà 33, 56127 Pisa, Italy; 2 Department of Excellence in Robotics&AI, Scuola Superiore Sant’Anna, Piazza Martiri Della Libertà 33, 56127 Pisa, Italy; 3 Dipartimento Di Ingegneria Civile E Industriale (DICI), Università Di Pisa, Largo Lucio Lazzarino 1, 56122 Pisa, Italy; 4 Centre for Neuroprosthetics and Institute of Bioengineering, School of Engineering, Bertarelli Foundation Chair in Translational Neuroengineering, ÉcolePolytechniqueFédérale de Lausanne (EPFL), 1007 Lausanne, Switzerland

**Keywords:** Carbon fibers, Conductive hydrogel, Neural interface, Bioelectronic medicine

## Abstract

Peripheral neural interfaces facilitate bidirectional communication between the nervous system and external devices, enabling precise control for prosthetic limbs, sensory feedback systems, and therapeutic interventions in the field of Bioelectronic Medicine. Intraneural interfaces hold great promise since they ensure high selectivity in communicating only with the desired nerve fascicles. Despite significant advancements, challenges such as chronic immune response, signal degradation over time, and lack of long-term biocompatibility remain critical considerations in the development of such devices. Here we report on the development and benchtop characterization of a novel design of an intraneural interface based on carbon fiber bundles. Carbon fibers possess low impedance, enabling enhanced signal detection and stimulation efficacy compared to traditional metal electrodes. We provided a 3D-stabilizing structure for the carbon fiber bundles made of PEDOT:PSS hydrogel, to enhance the biocompatibility between the carbon fibers and the nervous tissue. We further coated the overall bundles with a thin layer of elastomeric material to provide electrical insulation. Taken together, our results demonstrated that our electrode possesses adequate structural and electrochemical properties to ensure proper stimulation and recording of peripheral nerve fibers and a biocompatible interface with the nervous tissue.

**Supplementary Information:**

The online version contains supplementary material available at 10.1186/s42234-024-00154-5.

## Introduction

Neurological disorders and neuropathies result in significant problems for healthcare, impacting patient quality of life and presenting substantial challenges for treatment and management. These conditions alter the normal action potential transduction, resulting in impaired function of the innervated target organ. For patients, this means a loss of sensorimotor function in the limbs and potential dysfunction of internal organs, such as heart rate variability, difficulties in swallowing, and impeded gut peristalsis in case of vagus nerve damage. In recent years, Bioelectronic Medicine (BM) has represented an innovative paradigm for repairing the functionality of damaged organs by electrically stimulating or inhibiting the activity of a target peripheral nerve connected to the target organ (Pavlov et al. 2022). By applying a proper bioelectronic modulation protocol it is possible to restore a lost or compromised function by implanting an electronic device onto the nerve of interest, named Neural Interface (NI) (Cutrone and Micera 2019). In recent years, peripheral NIs have been successfully used to treat various conditions, restore the sense of touch in upper limb amputees (Raspopovic et al. 2014), support nerve reinnervation (Delgado-Martínez et al. 2017), and enable patients with spinal cord injuries to regain the use of lower limbs for assisted walking (Rowald et al. 2022). A class of NIs known as extraneural interfaces can be easily implanted by simply wrapping them around the nerve of interest, such as CUFF electrodes. These electrodes have been used to stimulate the cervical vagus nerve in patients to treat drug-resistant epilepsy (Orosz et al. 2014)and autoimmune diseases like rheumatoid arthritis (Koopman et al. 2016)and Crohn's disease (Bonaz et al. 2016; Bonaz 2021). Although successful, these devices are limited by their low selectivity for specific nerve fascicles, as they can only modulate the activity of superficial fibers, without accessing the deeper ones. This limitation prevents achieving the spatial selectivity needed to discriminate between various nerve fibers, thereby hindering precise neuromodulation that elicits a specific physiological function and increasing the risk of secondary effects on other organs (Paggi et al. 2021). For applications requiring high spatial selectivity, such as the modulation of fibers in the median, ulnar, and sciatic nerves, and for vagus nerve stimulation, intraneural stiff penetrating microprobes, such as the Utah (Wark et al. 2013)and Neuronexus (Wise et al. 1970)arrays, or intraneural polymer-based flexible arrays such as TIME electrodes (Boretius et al. 2010)provide greater precision (Badia et al. 2016; Normann 2016). Despite the success of such devices, their long-term stability is compromised by the tissue reaction against the implant, which triggers a cascade phenomenon called foreign body reaction (FBR) with the consequent establishment of chronic inflammation process and the fibrotic encapsulation of the device (Redolfi Riva and Micera 2021). These issues result in a considerable decline in the device's electrochemical performance and necessitate long-term use of anti-inflammatory drugs to prevent potential damage to nerve tissue from chronic inflammation. Consequently, a second surgical procedure is sometimes required to remove the electrode. For these reasons, a substantial demand for innovative and more biocompatible NIs is currently ongoing in the literature. Coating strategies with biocompatible hydrogels (Redolfi Riva et al. 2022; Moon et al. 2020; Huang et al. 2015), anti-fouling materials (Zou et al. 2021; Golabchi et al. 2019), or peptide functionalization to increase neuron cytocompatibility (Righi et al. 2018)are well-known strategies studied to improve the long-term integration of commercially available electrodes. Furthermore, recent literature has focused on the investigation of advanced NI designs, such as injectable mesh electrodes, featuring more biomimetic and conformable structures to enhance interaction with nervous tissue (Boys et al. 2023; Lee et al. 2019; Zeng et al. 2023). However, such strategies are currently limited to the research framework, leaving a significant gap between current devices and the clinical need for reliable NIs that enable long-term neuromodulation lasting months or years (Luan 2020; Carnicer-Lombarte 2021; Lotti 2017). Carbon fibers (CFs) based neural interfaces have raised the interest of the scientific community due to their excellent electrochemical properties, and microstructured dimensions that allow for good electrode/tissue integration. Specifically, compared to silicon-based microprobes with larger cross-sections, these microfiber electrodes have shown a diminished FBR after implantation with respect to traditional NIs (Hejazi 2021). They also offer a better signal-to-noise ratio for neural recording and superior resolution for neural stimulation. However, the mechanical properties of CFs still differ significantly from those of nerve tissue, leading to a mechanical mismatch that could damage the tissue in the long term. Moreover, the chemically inert nature of carbon poses challenges to surface functionalization and integration with more complex manufacturing processes to fabricate NIs (Devi et al. 2021). Furthermore, because of their shape, CFs suffer from tissue insertion problems, such as buckling (Thielen et al. 2021). A recent study found 2.5—3 mm as the maximum fiber length that could be inserted in vivo within brain tissue (Massey et al. 2019)and 1.1 mm within peripheral nervous system (Welle et al. n.d.; Jiman et al. 2020). Longer fibers would require external support to be inserted without buckling. This presents a disadvantage for intraneural NI applications, as penetrating the epineural tissue to reach the underlying nerve fascicles can be problematic. Moreover, the diameter of somatic human nerves could reach greater values (up to 10 mm for the ulnar nerve), leading to insufficient stimulation/recording selectivity. In order to overcome these limitations, our work presents a novel design of intraneural interface based on CFs bundle dipped within a conductive hydrogel matrix and further insulated with elastomeric materials. We refer to this device as intraneural bundle interface (IBI). Incorporating the conductive hydrogel helps reduce the mechanical mismatch between the CFs and nerve tissue, thereby improving long-term biocompatibility. Conventional NIs fabricated with metallic conductive traces can provoke adverse responses, such as fibrosis and scar formation in the target and surrounding tissues. These responses can lead to functional loss by decreasing the signal-to-noise ratio during recording and reducing the charge injection capacity during stimulation. To mitigate these issues, hydrogel interfaces have been introduced as adjuncts or alternatives to metallic electrodes (Yuk et al. 2022). Hydrogels offer enhanced biocompatibility due to their tissue-matching Young's modulus and lower bending stiffness, as well as improved electrical properties, including lower impedance and higher charge injection capacity (Yuk et al. 2019). Interposing a hydrogel layer at the electrode/tissue interface as*buffer layer*can effectively mitigate the mechanical stress at the tissue interface due to electrode micromotion, as confirmed by FEM modeling (Akouissi et al. 2022). Furthermore, a recent study supported hydrogel coating benefits by demonstrating that it could effectively reduce FBR effects on tissue, by reducing immune system activation and scar formation in a chronic 6-months rodents study (Park et al. 2021). We selected poly(3,4-ethylenedioxythiophene):poly(styrene sulfonate) (PEDOT:PSS) as the conductive polymer for the hydrogel, due to its excellent and well-known biocompatibility and conductive properties. The conductive polymer is processed using a unidirectional freeze-drying procedure to obtain a hydrogel network of aligned polymer chains that swells upon contact with a biological medium maintaining its conductive properties. We chose polydimethylsiloxane (PDMS) as elastomeric material to insulate the overall lateral surface of the electrode, exposing only its tip as active site for the stimulation/recording of the action potential. We performed morphological, physicochemical, and electrochemical characterization of our IBI to assess whether it would possess the features to correctly interface with a nerve structure and to be used as a neuromodulation device.

## Materials and methods

### Materials

PEDOT:PSS ((Clevios^TM^ PH1000, solid content 1.0 - 1.3 wt.%) was purchased from Heraeus Electronic Materials (Hanau, Germany). CFs having 7 µm average diameter and trimmed with 6 mm length were purchased from Goodfellows (Huntingdon, UK). PDMS was purchased as a two-part silicone elastomer kit from Dow Corning Corporation (Michigan, US). Phosphate buffer saline (PBS), DMSO, Sodium Alginate (MW = 80 kDa), Calcium Chloride anhydrous (CaCl_2_) and polylactic acid (PLA) were purchased from Merck (Darmstadt, Germany).

### Methods

#### PEDOT:PSS hydrogel preparation

**Fig. 1:**
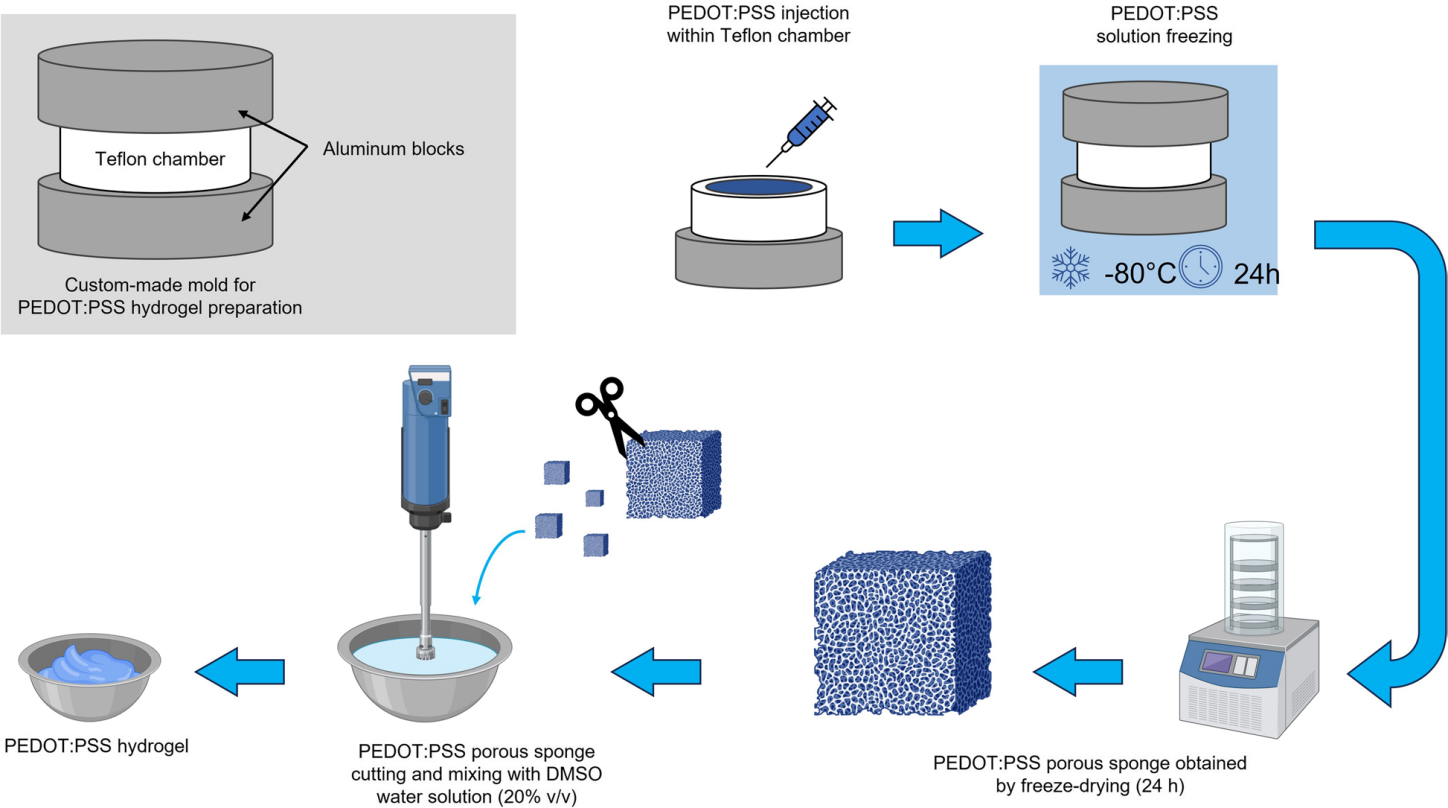
PEDOT:PSS hydrogel fabrication procedure. Created with Biorender.com

PEDOT:PSS hydrogel was prepared according to the method in (Lu et al. 2019) with some modifications and displayed in Fig.1.

A custom-made mold made by a cylindrical Teflon chamber and two aluminum blocks as caps was built to contain 10 mL of PEDOT:PSS solution and operate a unidirectional freezing procedure. After pouring the conductive polymer solution within the chamber, the mold was incubated at -80°C for 24 hours and freeze-dried for 24 hours to obtain a porous structure with longitudinally aligned microchannels. This structure was then cut with scissors, weighted, and homogenized in a water/DMSO solution (DMSO 20% v/v) for 1 minute to obtain a 3% (w/v) concentrated injectable hydrogel. This hydrogel was imaged with an optical microscope (HRX-01, Hirox, Tokyo, Japan) and printed with a 3D Bioplotter (EnvisionTEC GmbH, Gladbeck, Germany) or drop-cast onto to assess its physicochemical properties such as printability, water stability, and swelling. For swelling tests, a mold was printed using a Mega Zero 2.0 printer (Anycubic, China)) to deposit rectangular-shaped polymer films. The PEDOT:PSS solution was poured into the mold and cast at 80°C for 12 hours to obtain homogeneous films and then treated at 130°C for 30 minutes to perform thermal annealing. The films were immersed in a simulated physiological environment (PBS solution at 37°C) and weighted at different time intervals to calculate the swelling index (SI) with the following formula:

$$SI \left(\%\right)= \frac{{W}_{s}- {W}_{d}}{{W}_{d}}*100.$$

where W_s_ is the weight of the swollen film and W_d_ is the weight of the dry film.

#### IBI fabrication

**Fig. 2:**
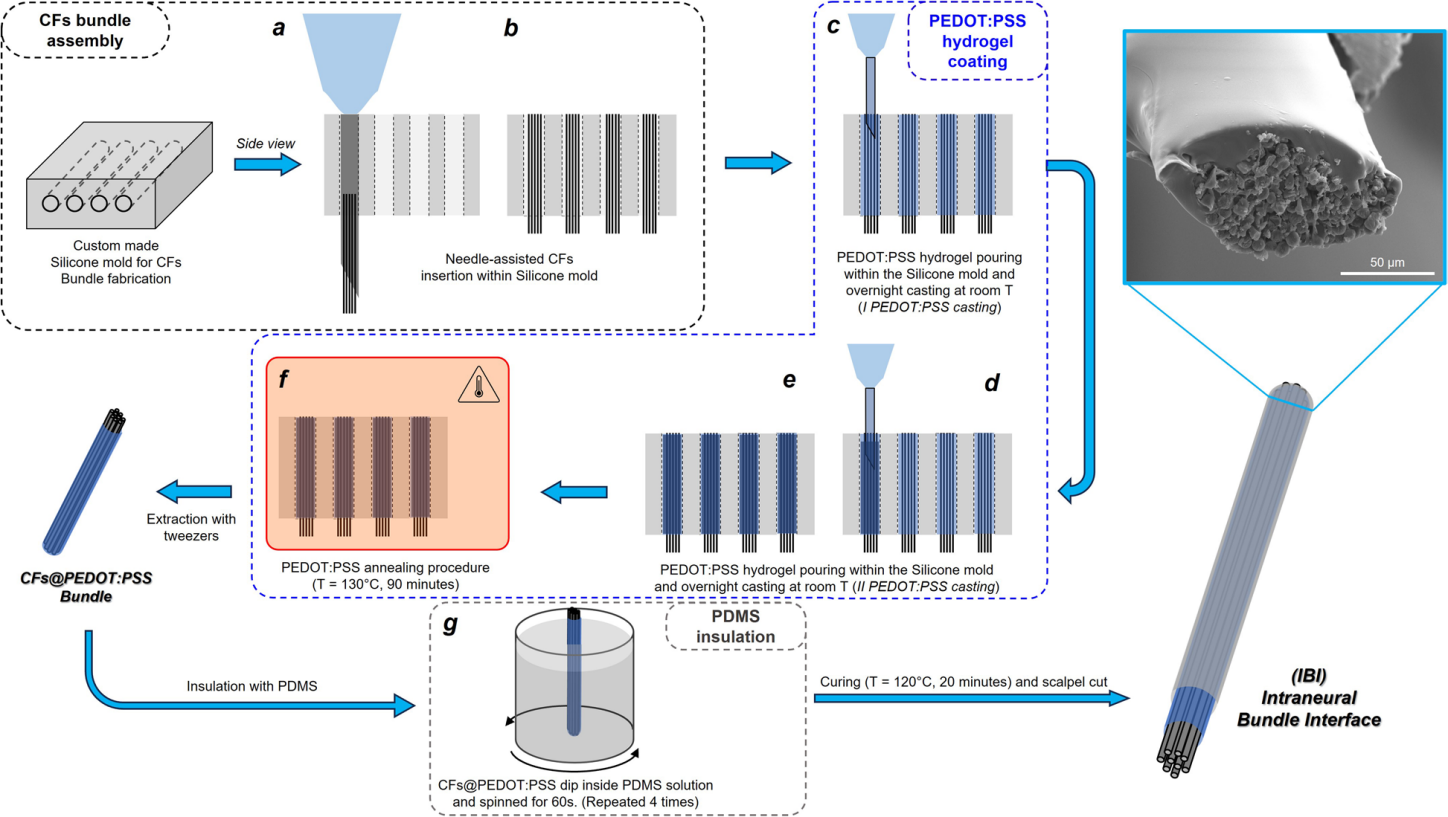
IBI fabrication procedure. Created with Biorender.com

IBI fabrication is composed by 3 different steps displayed in Fig. 2, namely CFs bundle assembly, PEDOT:PSS hydrogel coating and PDMS insulation.

##### CFs bundle assembly and subsequent PEDOT:PSS hydrogel coating

**Fig. 3:**
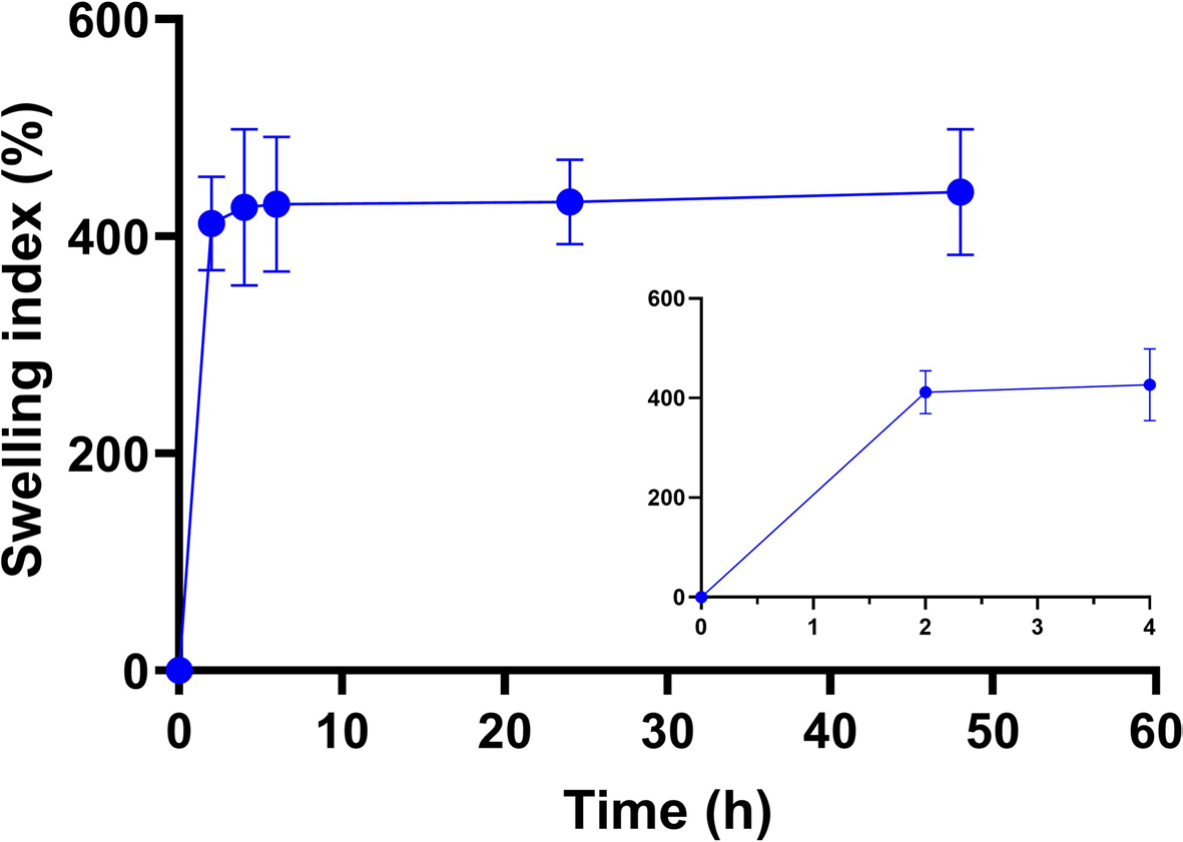
PEDOT:PSS hydrogel swelling dynamics in simulated physiological environment. The inset shows the depiction of the initial hours of the observation process

A custom-made Silicone mold patterned with microscale channels was prepared according to the method in (Song et al. 2010). Briefly, a PLA mold was printed with a FDM 3D-printer (UltiMaker S5, Geldermalsen, The Netherlands) and used to hold in parallel position 120 µm diameter metal wires. Silicone, prepared using a mixture of pre-polymer base and curing agent in a 9:1 volume ratio, was cast into the PLA mold and cured at 125°C for 25 minutes. After curing, Silicone was extracted from the PLA mold, and metal wires were removed obtaining a microscale channel patterned Silicone mold (Fig.2a). A 33-gauge needle was inserted into a Silicone mold channel, and a bundle of 6 mm long CFs was manually threaded inside the needle under the stereoscope (Vision Engineering Ltd, Woking, UK) until the entire cross-section of the needle was covered (Fig. 2b). A total of 240 CFs (d ≈ 7 µm) were used to fill the needle completely. The needle was then removed from the Silicone mold leaving the CFs bundle inside the channel. Using a second needle, the PEDOT:PSS hydrogel previously prepared was poured inside the Silicone mold and cast overnight at room temperature (first PEDOT:PSS casting, Fig. 2c). The PEDOT:PSS casting procedure was repeated a second time (second PEDOT:PSS casting, Fig. 3d). In order to facilitate DMSO evaporation and PEDOT:PSS polymeric chain rearrangement, the Silicone mold containing the PEDOT:PSS hydrogel and CFs was subjected to an annealing procedure at 130°C for 90 minutes (Fig. 2e). The CFs@PEDOT:PSS Bundle was then extracted from the Silicone mold with tweezers (Fig. 2f).

##### PDMS insulation

CFs@PEDOT:PSS Bundle was coated with PDMS to provide insulation to the overall lateral surface of the electrode and reduce the active site area. CFs@PEDOT:PSS Bundle was dipped in PDMS prepared using the same procedure described in Section “CFs bundle assembly and subsequent PEDOT:PSS hydrogel coating”, then subjected to spinning for 60 seconds to remove excess silicone and ensure layer uniformity. The PDMS-coated CFs@PEDOT:PSS Bundle was cured at 120°C for 20 minutes. This procedure was repeated 4 times (Fig. 2g). The PDMS-coated CFs@PEDOT:PSS Bundle tip was trimmed with a scalpel to expose the conductive tip, constituting the active site of the fabricated Intraneural Bundle Interface.

#### IBI characterization

To evaluate the contribution of the conductive PEDOT:PSS hydrogel, morphological and electrochemical characterization were performed on Control samples and IBI samples. Controls refer to PDMS-coated CFs Bundle without PEDOT:PSS hydrogel coating, while IBI refers to PDMS-coated CFs@PEDOT:PSS Bundle fabricated as described in “ IBI fabrication” Section. Scanning Electron Microscopy (SEM, Phenom XL, Eindhoven, The Netherlands) was used to morphologically characterize Control and IBI samples, evaluating their dimensions. Prior to SEM imaging, samples were coated with a thin layer of Au/Pd a few nm thick and mounted on metal sample holders using carbon tape. SEM images were obtained using secondary emission electrons (SED) modes scanning with 15 kV. Image processing and calculation were performed with Fiji (https://imagej.net/).

Electrode insertion dynamics were then evaluated using sodium alginate hydrogel as phantom nerve model. Briefly, a 2% (w/v) aqueous sodium alginate (Alg) solution was prepared and manually extruded within a saturated CaCl_2_ solution using syringes with 1 mm needle diameter. Upon contact with CaCl_2_, solution the extruded Alg solution undergoes instant ionotropic gelation (Sarmento et al. 2006). This hydrogel was kept for 30 minutes in incubation with CaCl_2_ solution and then for 3 hours in DI water to remove unreacted Ca^2+^ions, yielding worm-like structures with similar physiochemical properties to a peripheral nerve (Abdelbasset et al. 2022). Alg hydrogel were cut to the desired dimensions and used as phantom nerve model for IBI insertion studies, which were carried out using a tensile machine (Instron, USA) with custom setups. IBI samples were attached to a printed board circuit (PCB) that was clamped at the load cell of the tensile machine. A 0.25 mm/min constant speed was used to perform penetration test within the Alg hydrogel fixed onto a glass slide, measuring IBI insertion force versus displacement. The penetration depth was fixed at 1 mm (average thickness of the Alg hydrogel) and tests were manually stopped once a drifting in the insertion force was revealed (consistent with the electrode tip touching the surface of the glass slide holding the Alg hydrogel). Once penetration was completed, the electrodes were withdrawn and the puncture site on the hydrogel was analyzed with optical microscopy (HRX-01, Hirox, Tokyo, Japan) using 3D reconstruction tool.

Electrochemical impedance spectroscopy (EIS) and cyclic voltammetry (CV) were performed to evaluate the electrochemical properties of Control and IBI samples (n = 5). Prior to electrochemical analysis, samples were incubated in the electrolyte solution for 4 hours, to allow complete conductive hydrogel swelling. Measurements were obtained using a potentiostat (REF 600, Gamry Instruments, US) in a standard three-electrode configuration set-up in 1x PBS, with a platinum electrode as counter and an Ag|AgCl electrode as reference electrodes. EIS was performed delivering 5 mV AC waves from 1 MHz to 1 Hz. Average and standard deviation of 1kHz EIS values were evaluated and statistical analysis performed to compare the Control and IBI. CV was performed between −0.6 and 0.8V with a scan rate of 50 mV/s and the cathodic charge storage capacity (cCSC) was calculated as the time integral of the measured cathodic current. Average and standard deviation of cCSC values was evaluated and statistical analysis performed to compare Control and IBI. All the graphs were plotted using GraphPad Prism 8.

#### Statistical analysis

Data were expressed as mean ± standard error of the mean. Data were analyzed by one-way ANOVA followed by the Tukey posthoc test, using MATLAB R2021b software. Differences were considered statistically significant at *p* < 0.05.

## Results

Unidirectional freezing process yields 3D porous PEDOT:PSS sponges with highly longitudinally aligned microchannels (Figure S1a and S1b) resulting from the controlled nucleation of ice water crystals due to the temperature gradient guaranteed by the custom-made fabrication mold (Redolfi-Riva et al. 2023). This configuration ensures high PEDOT domains alignment within the microchannels. Subsequent hydration with a 20% (v/v) water/DMSO solution allows the obtainment of a hydrogel material thanks to interchain hydrogen bonding between PSS chains, due to the destabilization of the PEDOT-PSS interactions, with the formation of areas with high PSS^−^ chain packaging. At the same time, the formation of regions with high PEDOT^+^concentration allows high electron percolation, ensuring good electrical conductivity of the hydrogel (Wang et al. 2017; Xu et al. 2017). The hydrogel’s consistency allows for versatile fabrication for this formulation, which can be easily printed using additive manufacturing techniques (Figure S1c and S1d), extruded via nozzles and syringes, or drop cast to obtain microstructured films (Figure S1e). The hydrogel processed with water/DMSO solution displays good stability in the physiological environment, with consistent swelling that reaches stability after 2 h of incubation in PBS at 37 °C and maintains this configuration for 48 h of observation without significant variations (Fig.3). When handled, this hydrogel is soft, smooth, and flexible. Its maximum swelling after 48 h is 441 ± 58%, consistent with the behavior of hydrophilic hydrogels.

Contrarily, hydrogels processed without DMSO behave brittle and fragile and are not stable in water environment, as they tend to dissolve after a few hours of incubation in PBS. We deduced that this behavior is due to the absence of DMSO, which facilitates the formation of interchain hydrogen interactions among PSS^−^chains, thus preventing them from being solvated by water molecules. At the same time hydrogen bonding between the sulfonic acid groups in PEDOT and the polar groups of the DMSO additive improved charge-carrier mobility, leading to high conductivity in the PEDOT/DMSO structure (Lee et al. 2016). These results showed good physicochemical properties of PEDOT:PSS hydrogel and confirmed its adaptability to different manufacturing properties, thus allowing its incorporation as a building block of the IBI.

**Fig. 4:**
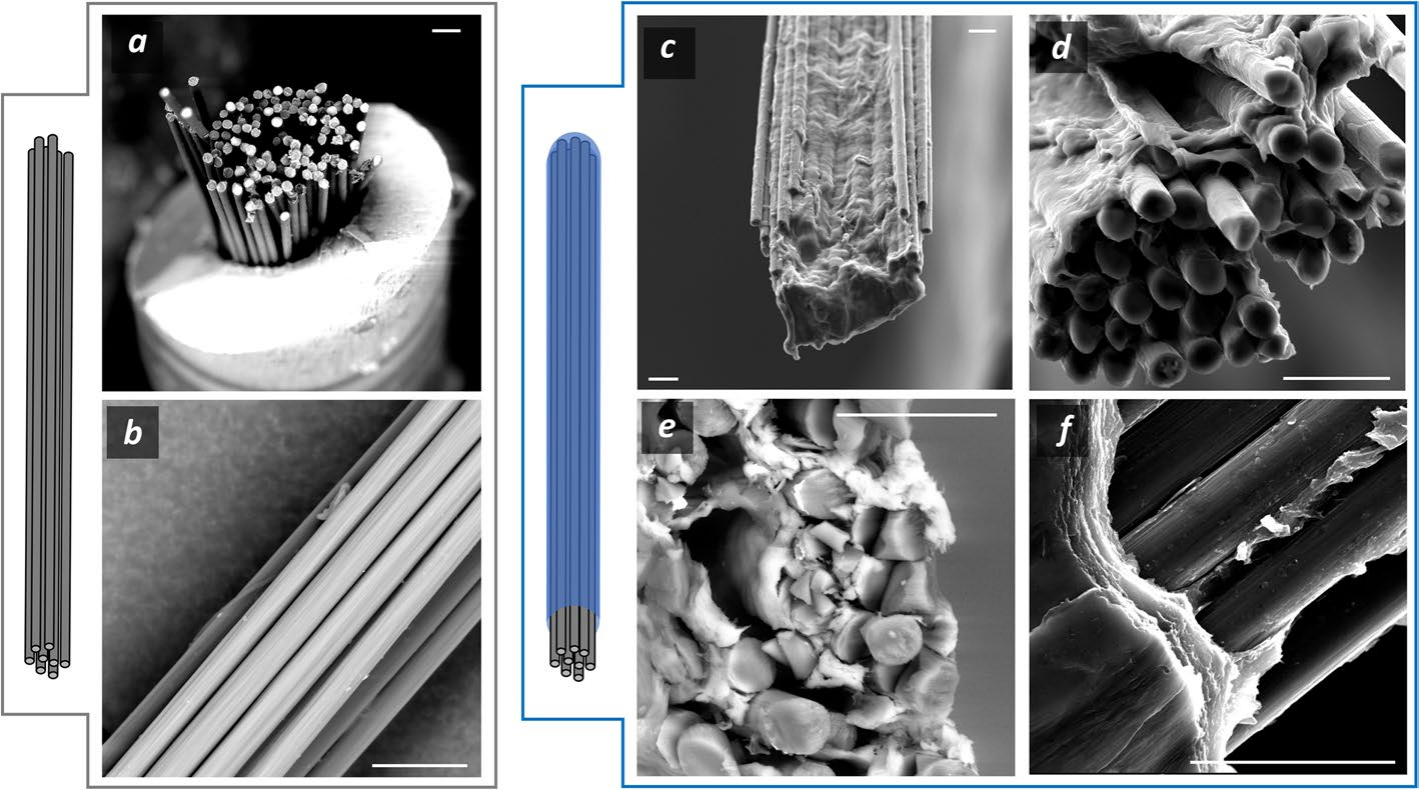
SEM images of CFs bundle loaded within the needle (**a**) and morphology of their lateral surface (**b**). PEDOT:PSS hydrogel coating of the CFs bundle (**c**,**d**) and magnification of the cross section (**e**) and lateral surface (**f**) of the coated CFs. Scale bars are 20 µm

SEM imaging of the IBI’s fabrication process shows successful PEDOT:PSS hydrogel coating of the CFs bundle (Fig. 4c-f).

CFs show a regular cylindrical shape with a very smooth surface (Fig. 4a and b) and were imaged using a needle to hold them together. Once PEDOT:PSS hydrogel was cast around the bundle, CFs permanently stick together forming a bundle without signs of hydrogel delamination or fiber detaching. The conductive hydrogel appears as a homogeneous layer that surrounds and holds together the CFs (Fig. 4c and d). Interestingly, the hydrogel does not only cover the CFs bundle but also penetrates within it interposing between the single CF fibers, thus acting as a 3D scaffold (Fig. 4e and Figure S2). This occurrence is responsible for the different bending behavior of the CFs bundle. Upon swelling under a water environment and when subjected to a bending force, the coated bundles behave as a single cohesive entity, deforming uniformly. In contrast, the uncoated bundles exhibit progressive CFs fraying under deformation due to the absence of a binding material to hold the fibers together (data not shown). Furthermore, no delamination of the structure was observed after stress application, supporting the proper integration of the two materials. These results confirm the correct PEDOT:PSS hydrogel coating of the CFs bundle.

**Fig. 5:**
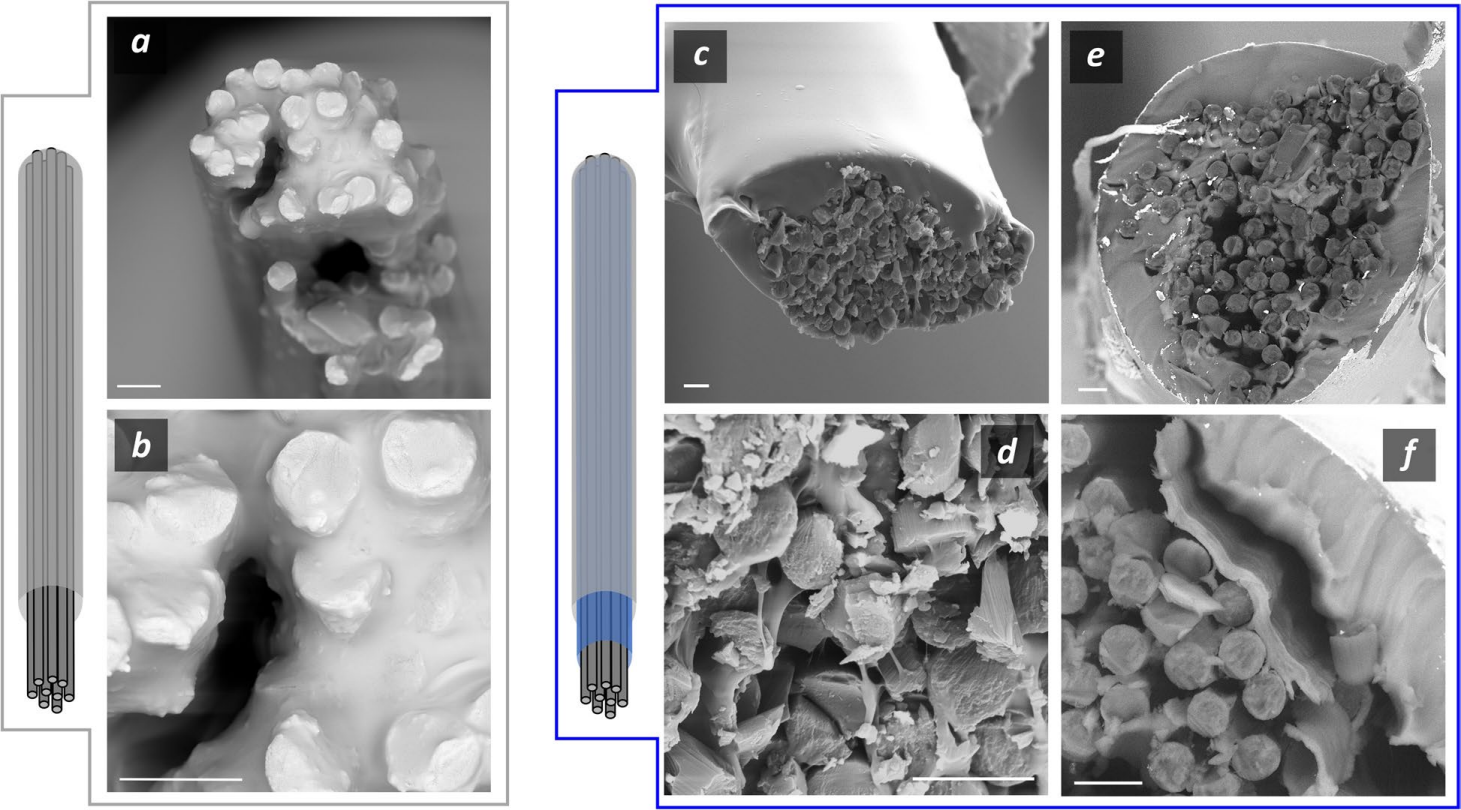
PDMS coating of CFs bundle (**a**,**b**) and CFs@PEDOT:PSS to yield the IBI (**c**,**e**). Magnification of PEDOT:PSS interpenetrating CFs bundle (**d**) and of the PDMS layer right on top of the PEDOT:PSS hydrogel layer (**f**). Scale bars are 10 µm

After conductive hydrogel coating, the material was covered with PDMS (Fig. 5) to insulate the device and a further cut step was operated with a scalpel to expose its tip to provide an active site for stimulation/recording of the nerve action potential (Fig. 5c-e). This latter procedure yields the final prototype of IBI.

PDMS coating results in a dense coating that surrounds the device with an average thickness of 27.2 ± 10.5 µm. Following this procedure, the final surface of the IBI differs markedly from the one that resulted from the previous conductive hydrogel coating step. It now features a remarkably smooth and uniform surface, characteristic of silicone materials, devoid of any asperities, with an approximately elliptical cross-sectional shape (Fig. 5c and 5e) with microstructured features displayed in Table 1. IBI dimensions are reported as major and minor axes of the ellipse fitting the cross-section of the device. It is worth noting that the PDMS coating integrates perfectly with the conductive hydrogel coating. The PDMS remains confined to the apical region of the device, positioned above the conductive hydrogel without infiltrating the fibers. This is facilitated by the presence of PEDOT:PSS scaffold, acting as a barrier, preventing PDMS from entering the CFs bundle (Fig. 5d and f). Conversely, when PDMS coats a CFs bundle lacking the hydrogel coating (Fig. 5a), the silicone layer permeates the bundle due to the absence of a protective material to preserve the carbon fibers (Fig. 5b).

**Table 1:** Geometrical features of the IBI during each fabrication step

	**Major Axis** **(µm)**	**Minor Axis** **(µm)**
CFs@PDMS (*Control*)	83.8 ± 12.6	60.9 ± 12.1
CFs@PEDOT	136,3 ± 47	71.1 ± 28.3
(CFs@PEDOT)@PDMS (*IBI*)	178.4 ± 25.6	132.4 ± 24.9

These results indicate that our manufacturing process effectively produces needle-like structures comprising three distinct materials with precise spatial confinement. The conductive hydrogel encapsulates and mechanically supports the CFs, forming a cohesive fiber bundle, while the PDMS layer envelops the electrode's lateral surface, ensuring electrical insulation.

To assess the capability of IBI to effectively penetrate nerve structure without damage, we performed insertion tests using an Alg hydrogel as nerve phantom model.

**Fig. 6:**
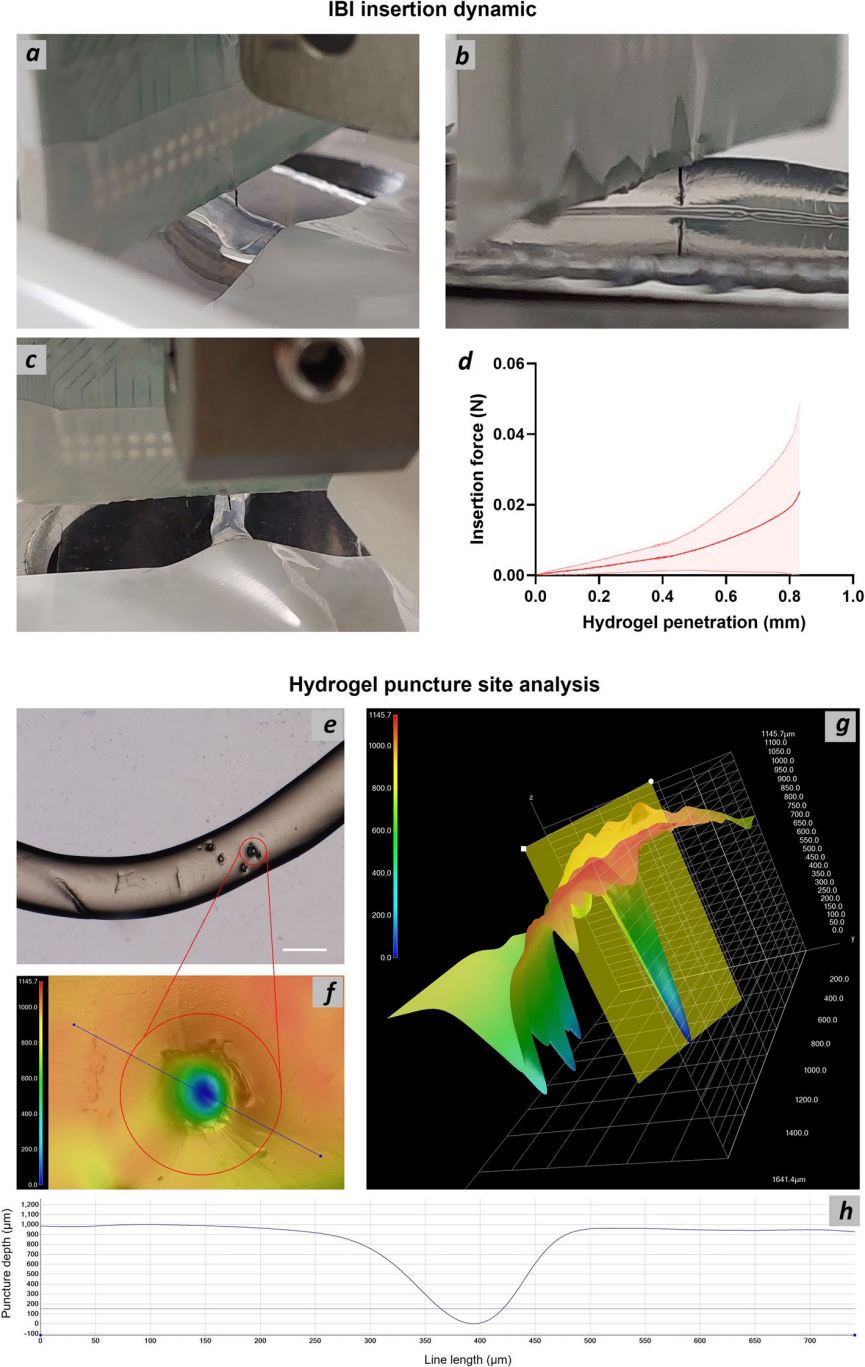
IBI insertion dynamics characterization. Photograph of IBI in contact with Alg hydrogel prior insertion test (**a**), at complete insertion (**b**) and after withdrawal (**c**). Plot of the average IBI insertion force vs hydrogel penetration (displacement) (**d**). SD are represented as dotted lines upon and below the average curve. Hydrogel puncture site analysis. Optical microscopy showing Alg hydrogel with multiple insertion areas (**e**). Scale bar is 1 mm. Magnification of a puncture site with color bar representing its depth (**f**) and plot of the surface profile relative to the blue line in (**f**) representing the depth of the puncture site (**h**). 3D reconstruction of the puncture site with color bar representing its depth

Insertion dynamics analysis showed that IBI can effectively penetrate (Fig. 6b) a nerve phantom and to be withdrawn from it (Fig. 6c) while maintaining its original structure without any sign of brake or CFs delamination, confirming stability of the design. Insertion force profile is in line with previous reports of silicon neural probes inserted within hydrogels (Li 2024) with a maximum value of 20 ± 19 mN at 0.8 mm hydrogel penetration (displacement), confirming good insertion dynamics with low stress on the IBI. Analysis of the puncture site showed effective IBI insertion of the Alg hydrogel, which was completely penetrated by IBI (Fig.6f, g and h).

Electrochemical characterization was performed to determine whether our electrode exhibits the requisite properties for application as a neural interface. Results of CFs@PEDOT@PDMS electrodes (IBI) were compared with PDMS-coated CFs (CFs@PDMS, Control) to assess whether the conductive hydrogel plays a role in the overall electrochemical performances of the electrode.

**Fig. 7:**
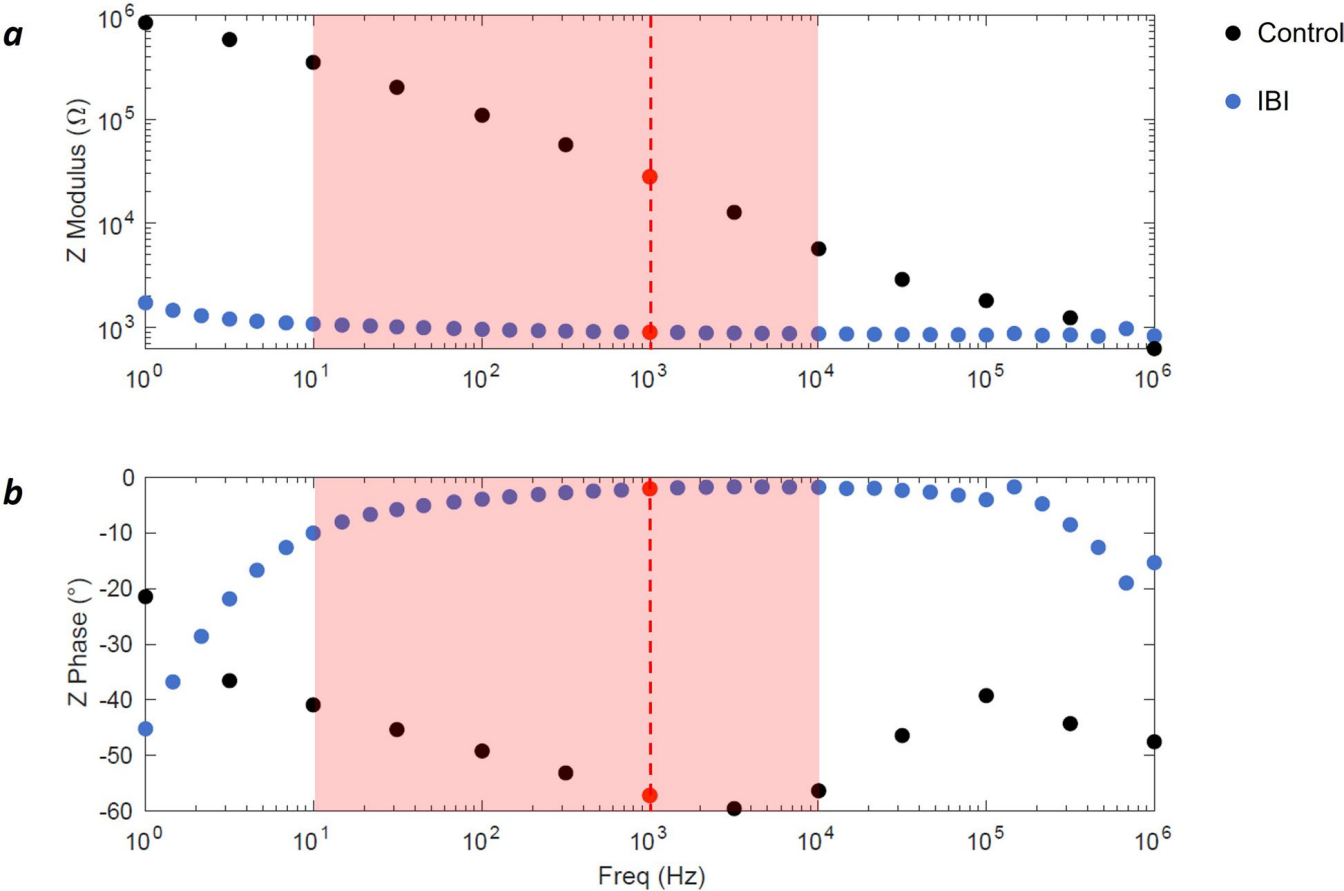
Electrochemical impedance spectroscopy (EIS) of IBI and control (CFs@PDMS) samples. Modulus (**a**) and Phase (**b**) of the electrodes’ impedance are reported as functions of the frequency in two different graphs. Significative frequencies for bioelectric phenomena are evidenced by a red box. A vertical red dashed line is reported to highlight the values at 10^3^ Hz, significative for neural recording

**Fig. 8:**
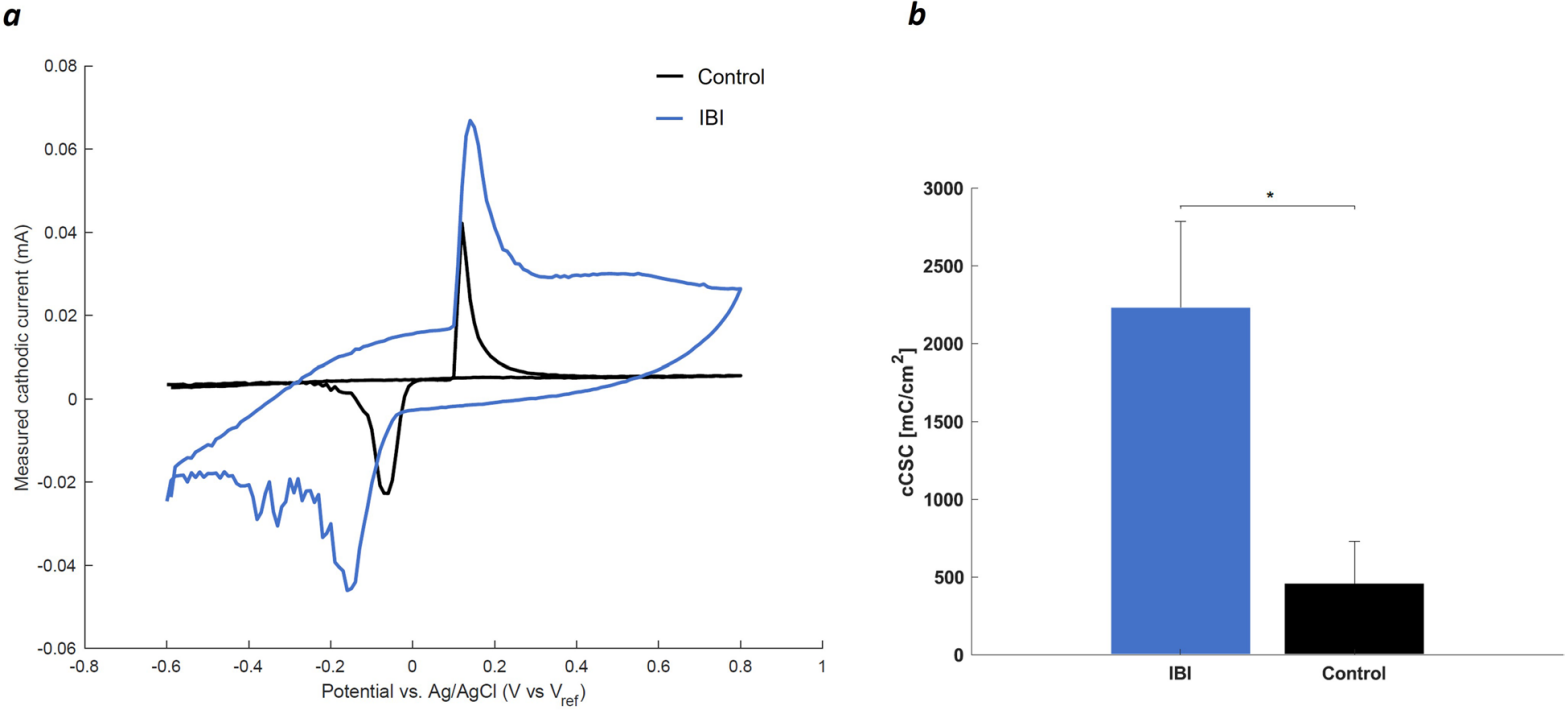
Cyclic voltammetry plot (**a**) and histogram reporting average cCSC values (**b**) for IBI and control samples (* *p* < 0.05)

Plot of the modulus of the electrode impedance shows marked differences between IBI and control samples. While control samples show capacitive behavior, IBI samples display a more resistive trend, resulting from the flatness of the curve (Fig. 7a). Interestingly, IBI samples show a drastic decrease of the impedance modulus at low frequency with respect to the control, with discrepancies of 1 and 2 orders of magnitude within the significative frequency interval for bioelectric phenomena (10 – 10^4^ Hz). In particular, at the frequency band of neuron action potential (1 kHz) IBI samples display a significative lower (*p* < 1^.^10^–4^) average impedance modulus of 1.79 ± 1.314 kΩ, while control samples display an average modulus of 20.67 ± 10.6 kΩ. Phase diagram shows a capacitive behavior at low frequencies (< 10 Hz) for IBI samples, that progressively tends to be more resistive, increasing within the significative interval for bioelectronic phenomena (Phase _IBI_@1 kHz = -8.9 ± 6.46°). Conversely, control samples show a significative (*p* < 0.05) lower phase within the whole 10 – 10^4^ Hz interval (Phase_control_@1 kHz = -54.9 ± 8.42°) (Fig. 7b). This phenomenon suggests an increase in electrode surface area for IBI samples with respect to control. We also evaluate the charge storage capacity of our electrode by performing CV measurements within physiologically relevant voltage from -0.6 to 0.8 V (Fig. 8).

CV plot shows clear redox peaks for both the IBI and control samples (Fig. 8a). PEDOT:PSS coating results in an increase of the absolute values and a shift of the cathodic and anodic peaks, which reflect a change in ions diffusion rate with respect to control samples (Cui et al. 2007). Importantly, the incorporation of the conductive hydrogel led to a remarkable increase in the area of the measured cathodic current for IBI samples with respect to control. In particular, a significative increase (*p* < 1.02^.^10^–5^) of cCSC for IBI samples is reported (Fig. 8b) (Fanelli et al. 2022).

## Discussion

In recent years, the application of NIs in the BM context has offered complementary therapeutic solutions alongside pharmacological treatments for various pathological conditions discussed in the Introduction. This development introduces new clinical therapeutic scenarios where bioelectronic and pharmacological approaches could be used in tandem, aiming to reduce drug dosages and associated systemic side effects. Furthermore, BM provides therapeutic options for patients unresponsive to pharmacological treatments, those for whom such treatments are impractical, or individuals who have developed sensitivities to the active principle (Koopman et al. 2017). This rationale underpins the growing interest in this technology and the efforts of the scientific community to make it reliable for long-term use. The novelty of this study lies in the integration of a conductive hydrogel formulation within an electrode composed of CF bundles insulated by PDMS, aiming to assess its potential use as an intraneural NI. To the best of our knowledge, this is the first report that describes such a design proposed as intraneural NI. We selected these materials for IBI fabrication due to their well-documented cytocompatibility and long-term stability after implantation in animals (Hejazi 2021; Green et al. 2015; Bianchi et al. 2022). The PEDOT:PSS hydrogel is processed through a manufacturing process that yields a stable and highly hygroscopic hydrogel with good rheological and conductive properties (Lu et al. 2019)that was previously proposed as soft conductive material for intracortical recording (Yuk et al. 2020)and recently by our group as the conductive material of fully-polymeric CUFF electrode (Zinno 2023). Its high adaptability to various manufacturing processes (Figure S1) allowed its integration as a building block of the IBI in this work. SEM imaging and morphological characterization of the conductive hydrogel demonstrate that this component envelops the CF bundle, acting as a 3D scaffold (Fig.4c-f) with a substantial swelling degree (SI ≈ 400%) when exposed to a simulated physiological environment. This effect is crucial as it ensures excellent ionic permeability, which is ideal for achieving a high signal-to-noise ratio during action potential recording (Yuk et al. 2019). Furthermore, the direct contact of a hydrogel material with the nervous tissue upon implantation respect to bare CFs ensures to minimize the mechanical mismatch between the two entities, thus lowering the FBR effect in chronic neuromodulation, while enhancing recording stability in long-term experiments thanks to its swelling dynamics. (Lu et al. 2019; Zhang et al. 2013; Lacour et al. 2016). Further elastomeric coating allowed proper electrical insulation with a well-known material for good integration with biological tissue, as it possesses similar mechanical properties with nerve tissue (Palchesko et al. 2012). Cutting procedure after PDMS coating provides the IBI with an elliptical-shaped active site, that exposes the PEDOT:PSS-coated CFs bundle with the nerve tissue surrounded by PDMS insulation. We tuned fabrication parameters to obtain active site dimensions ranging from 130 to 170 µm (Table1), in line with the anatomical configuration of important human somatic nerves, such as femoral (200 – 500 µm average fascicle cross-section) (Gustafson et al. 2009), ulnar (400 – 500 µm average fascicle cross-section) (Brill et al. 2017)and sciatic (300 – 500 µm average fascicle cross-section) nerve (Sladjana et al. 2008). This is peculiar, as designing NIs with active sites comparable to the average cross-sectional dimensions of the target nerve is one of the key aspects of selective neuromodulation, as it allows targeting the desired fascicle within the nerve structure (Yildiz et al. 2020; Larson et al. 2020; del Valle 2013). Moreover, it is reasonable to hypothesize that a structure with such morphology will safely penetrate peripheral nerve tissue without bucking, as evidenced by recent studies conducted with electrodes with comparable structural features (Welle et al. n.d.; Jiman et al. 2020; del Valle 2013; Yan 2019). In order to validate this hypothesis, we performed IBI penetration tests (Fig.6), and we demonstrated that our electrode design can successfully be inserted and retracted within hydrogel with similar physiochemical structure respect to peripheral nerve maintaining its shape with no sign of structural damage, confirming its possibility to be used as intraneural interface. Once it was established that the IBI has proper morphological characteristics to interface with a peripheral nerve, we conducted electrochemical characterization to confirm that the electrode possesses the appropriate properties for stimulating or recording nerve action potentials. EIS spectra evidenced a remarkable decrease in electrical impedance within physiologically relevant frequencies thanks to the conductive hydrogel coating (Fig. 7a). This effect is due to the enhanced surface-to-volume ratio provided by PEDOT:PSS coating of CFs that increases ionic permeability and guarantees a high signal-to-noise ratio for action potential recording (Yuk et al. 2019). It is worth noting that the average value of the impedance at 1 kHz for IBI samples is less than 2 kΩ, comparable with the value calculated for the transversal intrafascicular multichannel electrode (TIME) and 1-order of magnitude less than other well-known NIs, such as the self-opening neural interface (SELINE) and other electrodes based on soft polymers and conductive hydrogels (Table 2).

**Table 2:** Comparison of electrochemical performances of IBI with state-of-the-art NIs

	$\left|{\varvec{Z}}\right|$**@1 kHz (kΩ)**	**cCSC (mC/cm**^**2**^**)**	**Average active site area (mm**^**2**^**)**	**Ref**
TIME	6.2 ± 1.3	2.3	0.0028	(Boretius et al. 2010; Boretius 2012)
SELINE	88.08 ± 3.61	Not reported	0.0037	(Cutrone et al. 2015)
Electronic dura mater	5.2 ± 0.8	46.9 ± 3.3	Not reported	(Minev et al. 2015)
PEDOT:PSS intracortical NI	50–150 range	Not reported	0.001–0.003 range	(Yuk et al. 2020)
WPI CUFF electrode	2–5 range	Not reported	Not reported	https://www.wpi-europe.com/
IBI	1.79 ± 1.3	2.23^.^10^3^ ± 554.97	0.018	This work

The charge injection capacity of NIs must be adequate to depolarize the cell membrane beyond the action potential threshold to ensure successful neuromodulation. While the required charge injection varies among different types of neurons, small NIs necessitate a high charge injection limit (Pranti et al. 2018). This can be accomplished by reducing electrode impedance and by increasing the cCSC which consequently improves the quality of neural signal recording and stimulation, as shown by the incorporation of the conductive hydrogel within IBI structure. Further CV analysis revealed significative (*p* < 1.02^.^10^–5^) improvement of electrode charge storage for devices coated with conductive hydrogel that display enhanced capacitive effect thanks to the presence of PEDOT:PSS (Fig. 8). This effect is correlated to an increase of the electrochemical active area of the electrode, and is attributed to the increased surface area of the PEDOT:PSS hydrogel, which enables more effective diffusion of electrolyte ions at the electrode-solution interface, thereby enhancing cCSC of IBI (Zeng and Wu 2022; Dijk et al. 2020). Furthermore, during stimulation the conductive hydrogel surface was activated, resulting in an improved electron diffusion at the electrode–electrolyte interface respect to the control samples.

Overall, the results of this study confirmed that the IBI possesses the adequate requirements to correctly interface with the anatomy of a nerve or a neuronal structure of interest. Additionally, the electrochemical properties imparted by the conductive hydrogel confirm that our electrode exhibits the requisite characteristics for the successful stimulation or recording of nerve action potentials.

A potential application of the IBI involves its use as a penetrating active site for neuromodulation assembled within a high-density microelectrode array. This device would incorporate a specified number of IBIs, determined based on the fascicular anatomy of the target nerve, which penetrates the nerve to achieve selective recording and stimulation. Therefore, the IBI is suitable for use as an intraneural NI, enabling the monitoring of neural signals and the control of neuroprosthetic systems or the delivery of bioelectronic neuromodulation within the BM framework paving the way for its use in closed-loop applications, such as cardiac or bladder functions (Cracchiolo et al. 2021; Giannotti et al. 2023).

The specific structure of the IBI allows for future developments that could enhance its performance. The conductive hydrogel can be functionalized with molecules or drug delivery systems incorporating neurotrophins to improve cytocompatibility with Schwann cells. This, in turn, would facilitate the local reinnervation process following device penetration into the target nerve (Bianchini et al. 2023; Riva et al. 2024). Additionally, the conductive hydrogel can be enriched with nanostructures that function as nanotransducers for wireless neuromodulation, as described in (Micera et al. 2022). This utilization could lead to advancements in the long-term modulation of neural activity, contributing to enhanced patient outcomes in neuroprosthetic and BM interventions.

## Conclusions

This work presented a novel design of intraneural NI based on CFs bundle. Our characterization showed that our device possesses structural features compatible with peripheral nerve anatomy and good electrochemical performances, thus effectively modulating the activity of neuronal structures. We selected materials well-known for their biocompatibility. Specifically, the incorporation of the PEDOT:PSS hydrogel formulation allowed for a remarkable reduction of electrical impedance at the physiologically relevant frequency range and for significant improvement of the capacitive behavior, as shown by the increase of cCSC upon hydrogel incorporation. Another important contribution of the PEDOT:PSS hydrogel is to reduce the mechanical mismatch with the nerve tissue thus improving the long-term safety of the IBI. In conclusion, our study reported that the IBI has the potential to be used as intraneural NI for long-term neuromodulation. Future in vivo experiments will be needed to validate this hypothesis.

### Supplementary Information

Supplementary Material 1.

## Data Availability

Not applicable.

## Abbreviations

PEDOT:PSS: Poly(3,4-ethylenedioxythiophene) polystyrene sulfonate
BM: Bioelectronic Medicine
NI/Nis: Neural Interface/s
TIME: Transversal intrafascicular multichannel electrode
FBR: Foreign body reaction
CFs: Carbon fibers
IBI: Intraneural bundle interface
FEM: Finite element modeling
PDMS: Polydimethylsiloxane
PBS: Phosphate buffer saline
DMSO: Dimethyl sulfoxide
PLA: Polylactic acid
CaCl_2_: Calcium Chloride anhydrous
SEM: Scanning Electron Microscopy
SI: Swelling index
Alg: Sodium Alginate
PCB: Printed board circuit
EIS: Electrochemical impedance spectroscopy
CV: Cyclic voltammetry
cCSC: Cathodic charge storage capacity
SELINE: Self-opening neural interface

## Data availability

No datasets were generated or analysed during the current study.
