# Supplementary material for: Characterization of a conductive hydrogel@Carbon fibers electrode as a novel intraneural interface
Source: Bioelectron Med. 2024 Aug 27;10:20. doi: 10.1186/s42234-024-00154-5 (PMC11348655; doi:10.1186/s42234-024-00154-5)
Supplement: Supplementary file 1 — Supplementary Material 1. [file 42234_2024_154_MOESM1_ESM.docx]

Characterization of a Conductive hydrogel@Carbon fibers electrode as novel intraneural interface

*Alice Giannotti^1,2^, Ranieri Santanché^3^, Ciro Zinno^1,2^, Jacopo Carpaneto^1,2^, Silvestro Micera^1,4^ and Eugenio Redolfi Riva^1,2,^**

*^1^ The Biorobotic Institute, Scuola Superiore Sant’Anna, Piazza Martiri della Libertà 33, Pisa 56127, Italy*

*^2^ Department of Excellence in Robotics&AI, Scuola Superiore Sant’Anna, Piazza Martiri della Libertà 33, Pisa 56127, Italy*

*^3^ Dipartimento di Ingegneria Civile e Industriale (DICI), Università di Pisa, Largo Lucio Lazzarino 1, 56122 Pisa, Italy*

*^4^ Bertarelli Foundation Chair in Translational Neuroengineering, Centre for Neuroprosthetics and Institute of Bioengineering, School of Engineering, École Polytechnique Fédérale de Lausanne (EPFL), Lausanne 1007, Switzerland*

*Corresponding e-mail:* [*eugenio.redolfiriva@santannapisa.it*](mailto:eugenio.redolfiriva@santannapisa.it)

Keywords: Carbon fibers, conductive hydrogel, neural interface, bioelectronic medicine

**Supporting Information**


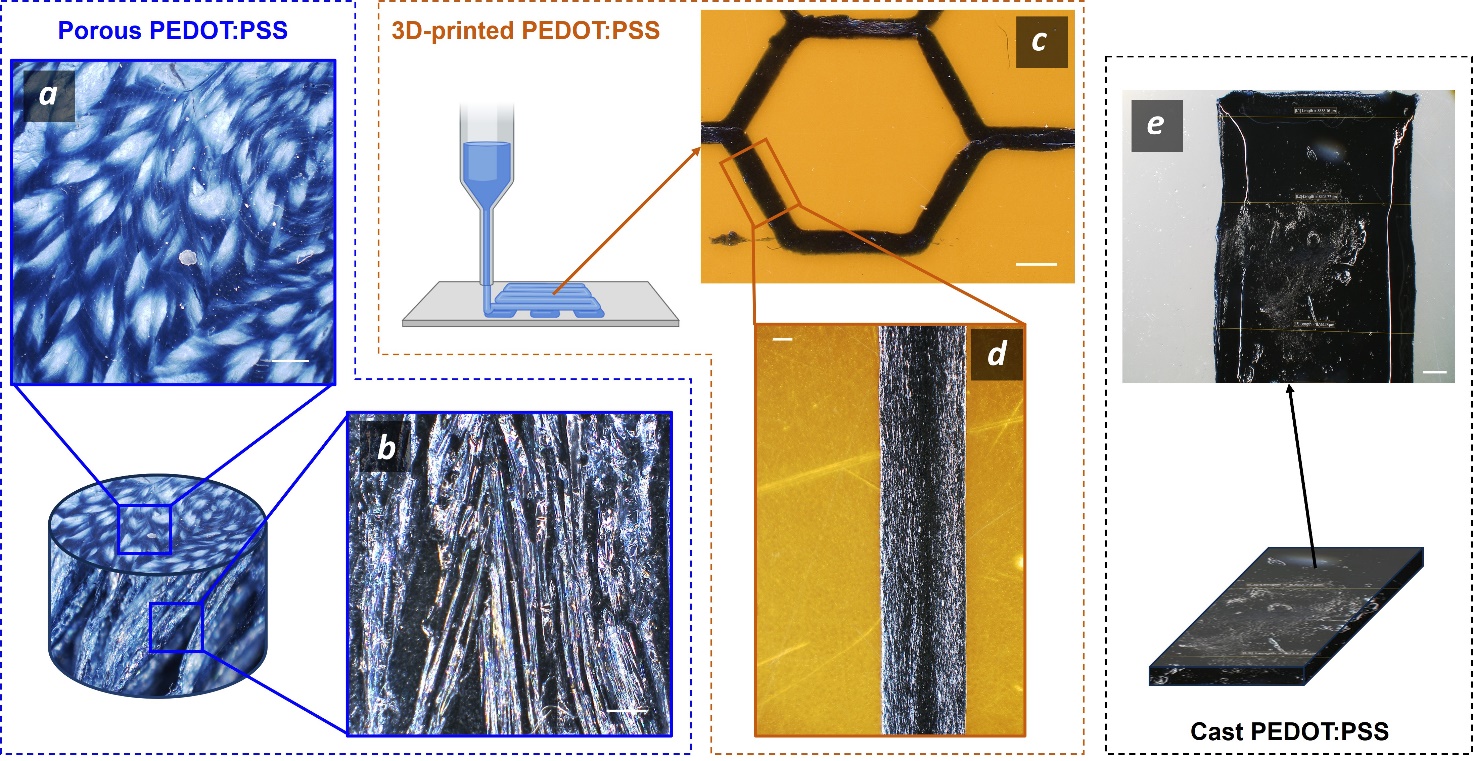


**Figure S1**. Photography of PEDOT:PSS hydrogel as porous sponge prior water/DMSO hydration (a,b), as 3D printed filament (c,d) and as drop-cast microstructured film. Scale bars are 1 mm (a, b, c and e) and 50 µm in (d).


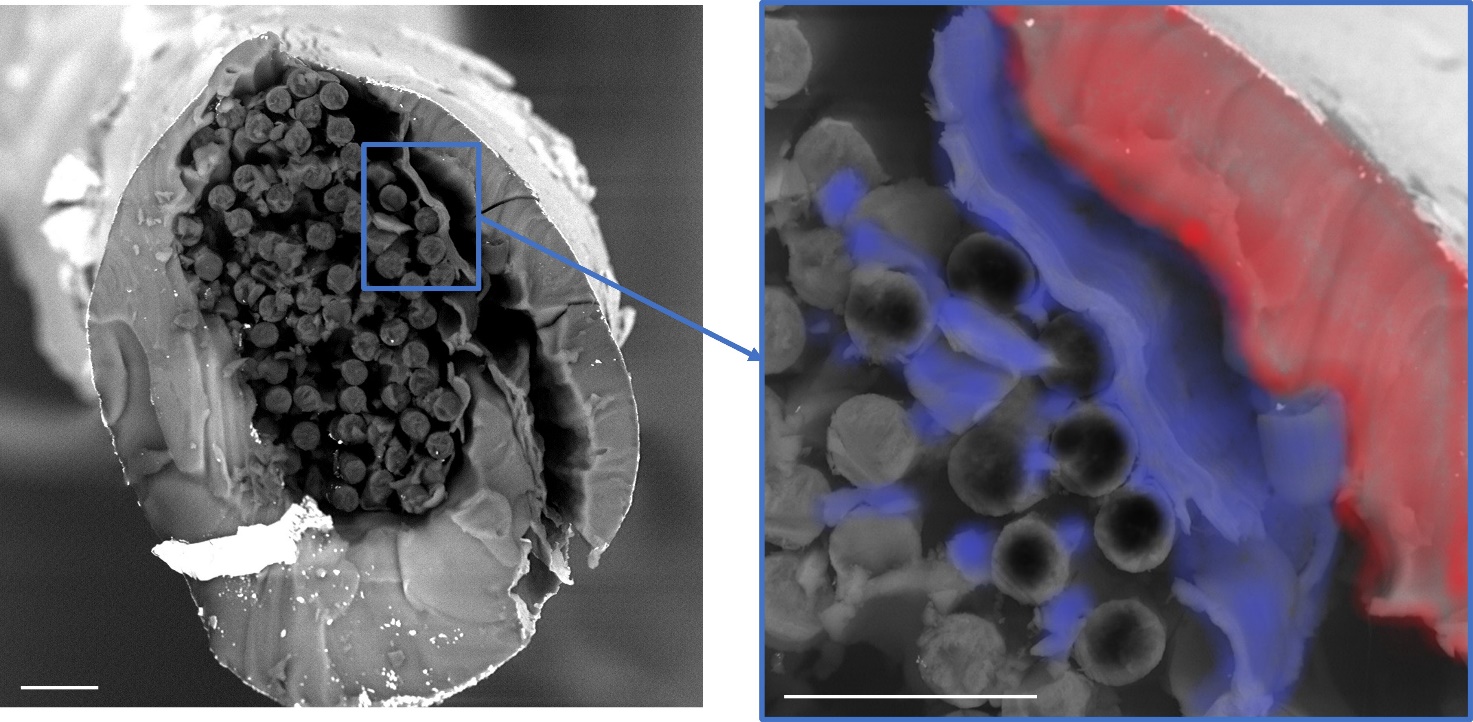


**Figure S2**. SEM images displaying the cross-section of IBI sample. In the inset, graphic representation of the correct spatial confinement between CFs (colored in black), the conductive hydrogel (colored in blue) and the elastomeric insulation (colored in red). Processed with Paint3D. Scale bars are 20 µm.
